# Cognitive efficacy and neural mechanisms of music‐based neurological rehabilitation for traumatic brain injury

**DOI:** 10.1111/nyas.14800

**Published:** 2022-06-08

**Authors:** Noelia Martínez‐Molina, Sini‐Tuuli Siponkoski, Teppo Särkämö

**Affiliations:** ^1^ Music, Ageing and Rehabilitation Team, Cognitive Brain Research Unit, Department of Psychology and Logopedics University of Helsinki Helsinki FI‐00014 Finland; ^2^ Centre of Excellence in Music, Mind, Body and Brain University of Jyväskylä & University of Helsinki Helsinki Finland

**Keywords:** brain morphometry, executive function, music‐based neurological rehabilitation, resting‐state networks, traumatic brain injury

## Abstract

Traumatic brain injury (TBI) causes lifelong cognitive deficits, most often in executive function (EF). Both musical training and music‐based rehabilitation have been shown to enhance EF and neuroplasticity. Thus far, however, there is little evidence for the potential rehabilitative effects of music for TBI. Here, we review the core findings from our recent cross‐over randomized controlled trial in which a 10‐week music‐based neurological rehabilitation (MBNR) protocol was administered to 40 patients with moderate‐to‐severe TBI. Neuropsychological testing and structural/functional magnetic resonance imaging were collected at three time points (baseline, 3 months, and 6 months); one group received the MBNR between time points 1 and 2, while a second group received it between time points 2 and 3. We found that both general EF and set shifting improved after the intervention, and this effect was maintained long term. Morphometric analyses revealed therapy‐induced gray matter volume changes most consistently in the right inferior frontal gyrus, changes that correlated with better outcomes in set shifting. Finally, we found changes in the between‐ and within‐network functional connectivity of large‐scale resting‐state networks after MBNR, which also correlated with measures of EF. Taken together, the data provide evidence for concluding that MBNR improves EF in TBI; also, the data show that morphometric and resting‐state functional connectivity are sensitive markers with which to monitor the neuroplasticity induced by the MBNR intervention.

## Graphical abstract

Here we review the core findings from our recent cross‐over randomized controlled trial (RCT) in which a 10‐week music‐based neurological rehabilitation (MBNR) protocol was administered to 40 patients with moderate‐to‐severe traumatic brain injury (TBI). The data provide evidence for concluding that MBNR improves executive function in TBI.

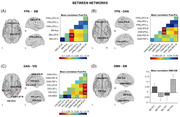

## INTRODUCTION

According to the U.S. National Institute of Neurological Disorders and Stroke, *traumatic brain injury* (TBI) is “an alteration in brain function, or other evidence of brain pathology caused by an external force.”^1^ Most of TBI cases are closed head injury and vary in severity from mild (including concussion) to moderate and severe, though this classification is fairly crude and generally based on a unidimensional assessment of level of consciousness^2^ or post‐traumatic amnesia.^3^ With considerable differences among high‐ and low‐ and middle‐income countries, the most common causes of TBI include falls and traffic‐related incidents.^4^, ^5^

Globally, TBI is a public health problem and leading cause of injury‐related death and disability.^4^ TBI is among the top three neurological conditions accounting for neurodisability globally, currently and in projections up to 2030 (see Ref. 6). The incidence of TBI varies by countries but recent reports provide an estimate of 50–60 million people with TBI per year worldwide.^7^ In the European Union, the age‐adjusted estimate for annual TBI approaches 2.5 million new cases.^8^, ^9^, ^10^ Owing in part to the demographic aging of the population in high‐income countries, incidence of TBI in the elderly, mostly due to falls, is increasing.^11^ Across all ages, TBI accounts for 30–40% of all injury‐related deaths.^12^ These figures not only highlight the magnitude of the problem but also the need to provide appropriate prevention and treatment strategies that are suitable for the subpopulations at higher risk of TBI.

People who suffer TBI show a wide spectrum of symptoms, ranging from physical, behavioral, emotional, to cognitive.^13^, ^14^, ^15^ This wide variation in the clinical manifestations of TBI is likely due to the complexity of the brain's organization, as well as to the patterns and extent of damage caused by external forces leading to TBI. Cognitively, the majority of deficits affect high‐level cognitive functions, such as attention, memory, communication, and executive function (EF).^16^ EF is a broad umbrella term referring to high‐level cognitive processes that enable individuals to regulate their thoughts and actions during goal‐directed behavior.^17^ Given their clinical importance and profound effect on the daily lives of the patients, executive dysfunctions are often viewed as a core symptom of TBI. While there is no consensus on the exact definition of EF, various cognitive processes, such as *set shifting* (switching from one task to another), *inhibition* (avoiding a prepotent response), and *updating* (continuously updating the contents of working memory), have been proposed as key components of EF.^18^

A brain hallmark of TBI, compared with other neurological disorders (e.g., stroke), is that the underlying brain injury is difficult to measure clinically. Computed tomography and standard structural magnetic resonance imaging (MRI) acquired in the clinical setting are most sensitive for measuring focal injuries in the form of microbleeds^19^ or hematomas,^20^ though these are not always present in individuals with TBI and, if not found in given cases, may lead to the underestimation of the severity of the injury. In addition to focal brain damage, rapid acceleration and deceleration forces at the time of brain injury damage the axonal membrane, which can disrupt axonal transport; such so‐called *diffuse axonal injury* (DAI)^21^, ^22^ is a key and common hallmark in the pathophysiology of all TBI severities. As mentioned, the detection of DAI poses challenges due to the lack of sensitive and low spatial resolution of current neuroimaging technologies—though some diffusion metrics, such as a neurite density index, could represent a more specific measure of the neurodegeneration and demyelinization caused by DAI.^22^

Given the diffuse structural disconnection triggered by DAI, it is relatively straightforward to predict the coexistence of functional connectivity (FC) abnormalities in large‐scale networks.^23^ Despite the complexity of the effects of TBI on network function, there are numerous studies showing the consistent patterns of functional network disruption,^23^ especially affecting the default mode,^24^ the salience, and the executive control networks,^25^ which suggests that the understanding and characterization of this neurological disorder may be greatly advanced from the study of static and dynamic brain interactions at the global level with the potential to be used as diagnostic or prognostic markers and readily implemented with current resting‐state functional magnetic resonance imaging.

Although TBI is usually limited to a single event, it has been shown that it confers an increased long‐term risk for cognitive impairment and dementia,^26^, ^27^ stroke,^28^, ^29^ parkinsonism,^30^, ^31^ and epilepsy,^32^ as well as being associated with a higher long‐term mortality rate compared with rates for the general population.

Taken together, these facts illustrate that TBI can have long‐term consequences for the patients, and that despite some degree of recovery, patients might have to continuously adapt and cope with any remaining symptoms. In this regard, the optimization of rehabilitation methods that are cost‐effective and can be tailored to the individual needs of patients may provide great benefits in terms of both reducing the economic impact to society and health care systems and improving clinical outcomes.

## MUSIC THERAPY FOR TBI REHABILITATION

The diversity and complexity of the consequences of TBI are best addressed with a comprehensive, holistic approach to rehabilitation delivered by a specialized multidisciplinary team, in close liaison with the patient and family or caregivers (the patient‐centered care approach).^33^ As recently noted,^34^ music therapy responds well to this framework and can take the shape of an active or passive intervention, a decision that must be made based on the primary clinical outcomes targeted by the intervention, as well as the severity of the symptoms presented by the patients. This becomes markedly clear when the stage of the recovery is considered. For instance, when a music therapy is provided to TBI patients with a minimally conscious or comatose state, a passive music listening intervention may be the most suitable strategy and help to regulate arousal and moderate physiological parameters.^35^ Music listening has been proven to ameliorate cognitive symptoms—including attention and verbal memory—in stroke patients at the acute stage.^36^ One possibility is that the neurobiological mechanisms underlying these effects may result from the widespread engagement of brain regions during music listening, which, in turn, may induce experience‐dependent plasticity to support lost or impaired brain function. Another important benefit derived from music listening is self‐regulation of emotions, as it recruits the reward system and an extensive number of limbic and paralimbic regions.^37^, ^38^ It then follows that music can be used to regulate mood alterations after a brain injury as has been shown in stroke patients.^36^ In addition, since music listening can act as a reinforcer on its own,^37^ it may contribute to the adherence to the therapy as opposed to traditional rehabilitation methods.

On the other hand, active music making by instrument playing or singing leads to further activation of the brain, requiring the precise timing, sequencing, and spatial organization of actions coordinated by the cerebellum, basal ganglia, premotor and supplementary motor areas, and prefrontal cortical regions.^39^ Importantly, behavioral and neuroimaging studies in healthy individuals have revealed that musical training enhances EF and the recruitment of the cognitive control network,^40^, ^41^, ^42^, ^43^, ^44^, ^45^, ^46^, ^47^, ^48^ raising the question of whether music‐based interventions could have similar positive effects on the executive dysfunction experienced by TBI patients. In spite of the evidence suggestive of music therapy being suitable for TBI rehabilitation, little clinical research has been conducted thus far.

A review of the literature reveals that only a few studies have investigated the clinical efficacy and neural correlates of music therapy after brain injury. In one of the earliest, Thaut and colleagues^49^ carried out a pre‐ and post‐test exploratory study with a treatment (*n* = 31) and control group (*n* = 23) of patients, with the latter receiving no intervention. The music therapy consisted of four 30‐min sessions each one targeting a different domain: attention, memory, EF, and emotional adjustment. The pre–post comparison suggested that treated patients improved in the mental flexibility aspect of EF, in addition to better self‐efficacy and reduced anxiety and depression.

The next effort in the literature is the small‐scale feasibility study conducted by Lynch and LaGlasse.^50^ The patients (*n* = 14) were randomly assigned to a music therapy group, singing group, and control group. The primary outcome of this musical executive function training (MEFT) intervention was the improvement of independent task switching. The MEFT was delivered in 5 consecutive days with a 1‐h session duration. The authors demonstrated the feasibility of this protocol for larger samples and found that the MEFT group improved in mental flexibility.

More recently, Vik *et al.* ^51^, ^52^ carried out a piano training intervention in a group of seven mild TBI (mTBI) patients who received two 30‐min one‐on‐one piano lessons per week for 8 weeks and, in addition, had to practice at home for a minimum of 15 min a day. The piano‐training protocol involved reading musical notation of 28 pieces for beginners. The study design also comprised two control groups of healthy participants, one with the music intervention (*n* = 11) and one without (*n* = 12). The results from the neuropsychological assessment indicated that verbal learning improved in the patient and control group with the music intervention. Furthermore, the authors found an increased activation of the right orbitofrontal (OFC) cortex in a tonic‐dominant‐tonic task in the mTBI patients after the intervention. Using a liberal threshold, seven regions of interest derived from this analysis were used as nodes in a spectral dynamic causal model.^52^ When compared to the control group that received the intervention, the mTBI patients showed increased intrahemispheric FC between the left OFC and reduced connectivity between the right anterior OFC and the left posterior OFC. The results from these analyses need to be replicated in a larger sample of mTBI patients but, as noted by the authors, might suggest that the music training induced plastic changes in the activity and connectivity patterns of the OFC, a region in close proximity to bony protrusions that is particularly vulnerable to trauma‐induced rotational acceleration of the brain. However promising, these findings must be taken with caution, as most of them include important limitations, such as small sample sizes, lack of a high‐quality randomized controlled trial (RCT), heterogeneity in the brain injury mechanism, and no brain imaging measures (except for Vik *et al.*’s work). To illustrate this, Thaut *et al.* ^49^ included not only patients with TBI but also some with stroke, toxic exposure, seizures, or brain tumor. In addition, there were differences in the number and severity of brain injuries between the control and intervention groups. Likewise, Lynch and LaGasse^50^ included one patient with stroke, the severity of which was not reported. Another important limitation concerns the outcome measures used in the aforementioned studies, which were brief and did not cover different domains of EF and focused attention systematically. Thus, there is a serious need for large RCT with a music‐based intervention that can provide robust translatable evidence for TBI rehabilitation.

## A LARGE‐SCALE RCT USING MUSIC THERAPY FOR TBI REHABILITATION

Given the nature and limitations of the prior studies for the efficacy of music‐based neurological rehabilitation (MBNR) in TBI, we sought to address some of them with the first‐ever large‐scale RCT in moderate‐to‐severe TBI.^53^, ^54^, ^55^ In this single‐blinded RCT, we used a cross‐over design with *n* = 40 TBI patients randomly allocated to two groups, AB and BA. The randomization was stratified for lesion laterality (left/right/bilateral) using an online random number generator. During the 3 months between the first two time points for data collection, the AB group (*n* = 20) received the 10‐week music intervention in addition to standard care, and vice versa for the second 3‐month period spanning data collection. The standard care included mostly individual therapies, such as physiotherapy, occupational therapy, neuropsychological rehabilitation, and speech therapy, provided by the health care system. Both groups received a similar amount of other rehabilitation (e.g., physical therapy or neuropsychological rehabilitation) at any time point (for more details, see Table 2 in our previous publication^53^).

For the purpose of the present study, we administered an MBNR intervention specifically designed to target the needs of TBI patients. The intervention model was adapted from two existing music therapy methods: functionally oriented music therapy (https://www.fmtmetoden.se/fmtsiteng/index.html) and music‐supported training, which have been both applied in stroke rehabilitation.^56^, ^57^ Our approach was centered on supporting neurological and cognitive recovery after TBI, as a distinction from other types of music therapy that might focus more, for instance, on emotional aspects. The primary outcome of this RCT was the rehabilitation of cognitive deficits, especially EF, attention, and working memory. Secondary goals were to enhance mood, emotional adjustment, and upper extremity motor function.

**FIGURE 1:**
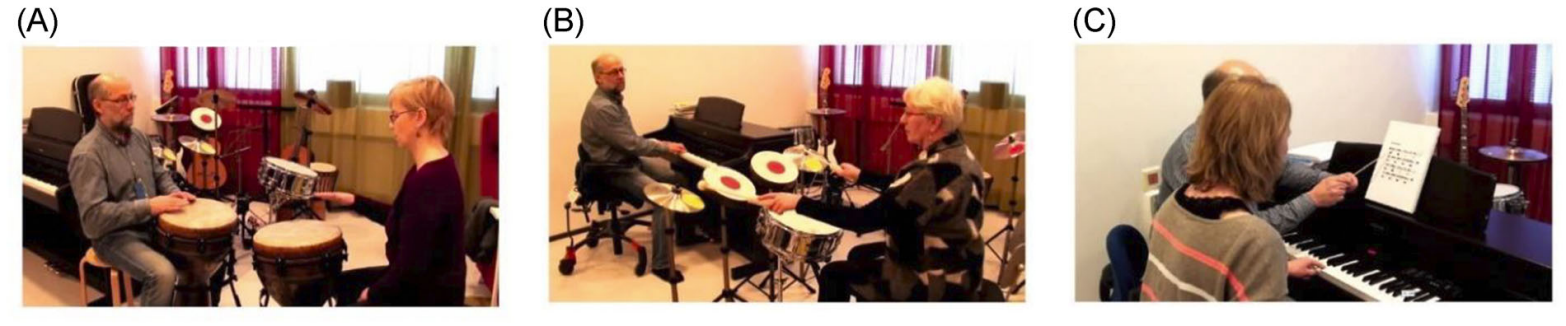
The three constituent modules of the music‐based neurological rehabilitation. (A) Rhythmical training with djembe. (B) Structured cognitive‐motor training with drum set. (C) Assisted music playing using figure notes

**FIGURE 2:**
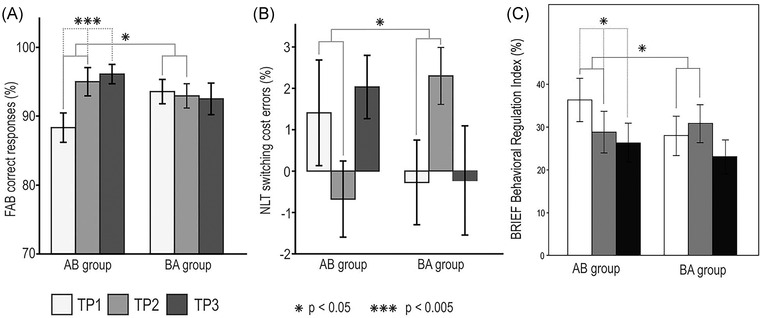
Behavioral test results from the intention‐to‐treat (ITT) analysis. (A) Frontal Assessment Battery (FAB) score, (B) Number–Letter Task (NLT) switching cost error (panel taken from Ref. 53), and (C) BRIEF‐A Behavioral Regulation Index (panel taken from Ref. 54). The bar plots (mean SEM) show changes in test scores over the three time points (TP) presented group‐wise (AB/BA) from the imputed data set (depicting the mean of 20 imputations). Significant time–group interactions are shown with solid gray lines, and significant within‐group time main effects are shown with dashed gray lines. Abbreviation: SEM, standard error of the mean

**FIGURE 3:**
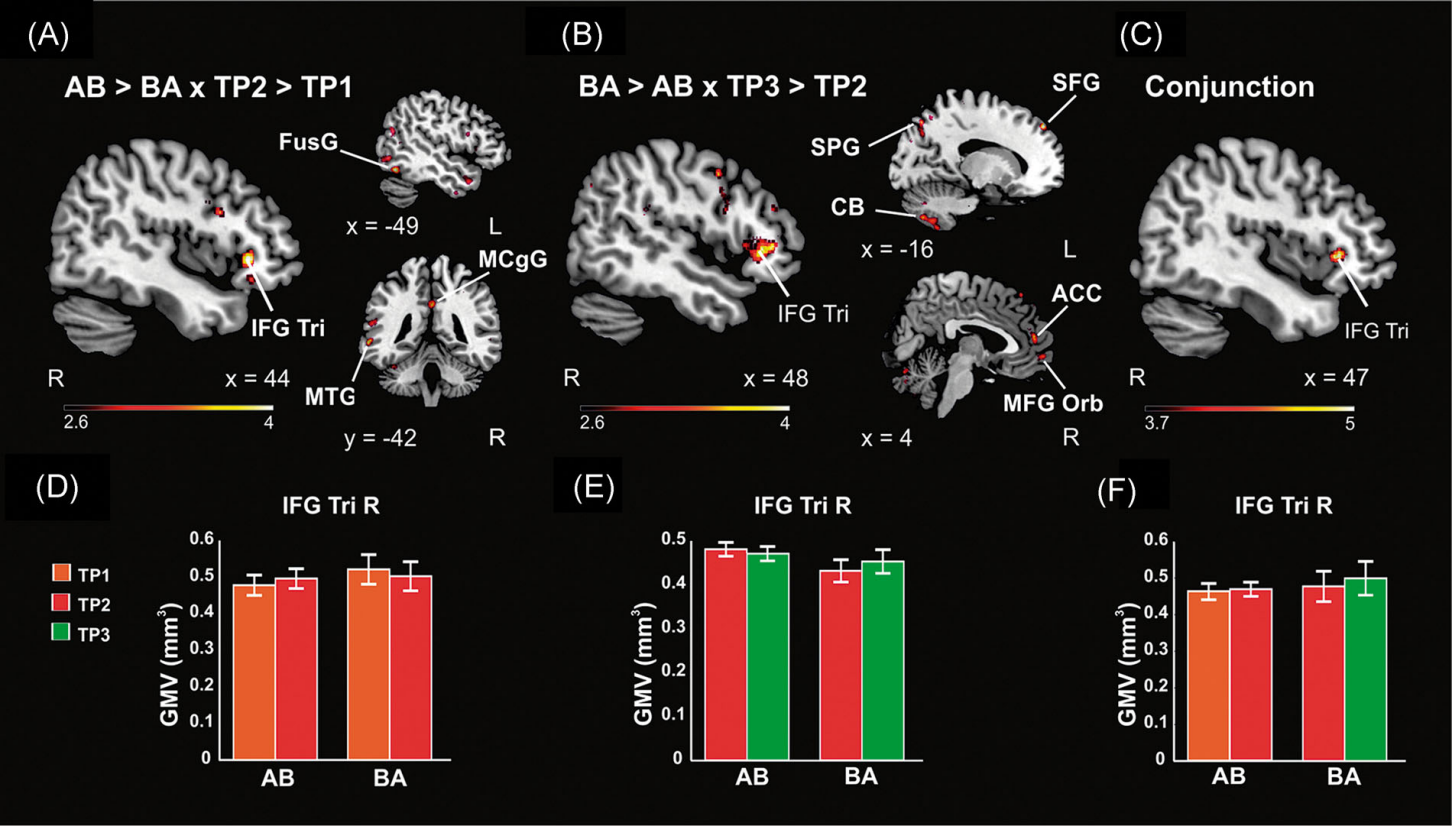
VBM results from the mixed‐model ANOVA, including 25 patients with TBI and three time points (TP). (A) Group (AB > BA) Time (TP2 > TP1) interaction in gray matter volume (GMV). (B) Group (BA > AB) Time (TP3 > TP2) interaction in GMV. (C) Conjunction analysis between A and B in GMV. (D–F) Bar plots (mean SEM) for GMV from the local maxima in the right IFG Tri cluster in A, B, and C contrasts, respectively. The results are reported in MNI coordinates and at an uncorrected *p* < 0.005 threshold at the voxel level for visualization purposes. Abbreviations: ACC, anterior cingulate gyrus; CB, cerebellum lobule 8; FusG, fusiform gyrus; IFG Tri, inferior frontal gyrus triangular part; L, left; MCgG, middle cingulate gyrus; MFG Orb, middle frontal gyrus orbital part; MNI, Montreal Neurological Institute; MTG, middle temporal gyrus; R, right; SEM, standard error of the mean; SFG, superior frontal gyrus; SPG, superior parietal gyrus; TBI, traumatic brain injury; VBM, voxel‐based morphometry. Figure taken from Ref. 53

The intervention consisted of 20 individual therapy sessions (2 times per week, 60 min per session) held by a trained music therapist. The length, frequency, and total duration (10 weeks) of the intervention were selected to balance training intensity with maintaining participant motivation and endurance, as well as logistic practicalities (e.g., traveling to sessions). The focus was on active music production with different instruments, and each session was structured in three 20‐min modules: (1) rhythmical training (Figure 1A), (2) structured cognitive–motor training (Figure 1B), and (3) assisted music playing (Figure 1C). Rhythmical training involved playing sequences of musical rhythms and coordinated bimanual movements using hands on a djembe drum and body percussion. Structured cognitive–motor training involved playing musical exercises on a drum set with varying levels of movement elements and composition of drum pads and drumsticks, with piano accompaniment provided by the therapist. Assisted music playing consisted of learning to play the participant's own favorite songs on the piano with the help of the therapist and using figure notes (https://www.figurenotes.org/what-is-figurenotes), a special musical notation system using colors and shapes originally developed in Finland, which makes music playing easily accessible without prior musical education.

During all training, the therapist provided a visual and auditory model, and the participants were encouraged to make their own observations of their playing. When the participants were able to produce a certain rhythmical entity or melody, the therapist would add accompaniment. Emphasis was on musical, nonlinguistic communication, in which the auditory feedback from the therapist was essential. Additionally, playing familiar songs on the piano using figure notes provided an intuitive, direct form of feedback for the participants. The intervention did not require prior musical training, and the difficulty level of the exercises was initially adjusted and increased in a stepwise manner within and across the sessions to meet the skill level and progression of the individual participant. Further, musical improvisation was included in all modules and encouraged throughout the therapy to provide a means of emotional expression. This was implemented through encouraging the participant to create individual playing styles and techniques of drumming and creating own rhythms and melodies, while the therapist provided a safe background through playing, for example, beat or chord progression. The therapist also adjusted the accompaniment to the musical ideas expressed by the participants to reinforce and validate them. The participants took part in choosing their favorite music for the sessions and level of performance, adjusted dynamics, tempo, and rhythm according to their individual style.

Baseline measurements were administered at time point 1 (TP1), and follow‐up measurements were conducted at the 3‐month cross‐over point (TP2) and 6‐month completion point (TP3). These measurements comprise an extensive neuropsychological battery and questionnaires, in addition to structural and resting‐state functional magnetic resonance imaging. Self‐report and caregiver‐report questionnaires, as well as subjective feedback, were also collected 18 months after the intervention. In the following, we will summarize the main findings from the analyses of this data set.

## EFFECTS OF MBNR ON COGNITIVE, BEHAVIORAL, AND EMOTIONAL RECOVERY AFTER TBI

The primary outcome was change in performance in the frontal assessment battery (FAB).^58^ FAB is a measure of global executive functioning and consists of six subtests covering different aspects of frontal lobe functions: conceptualization, mental flexibility, motor programming, sensitivity to interference, inhibitory control, and environmental autonomy. Furthermore, computerized tests were administered to capture more narrowed aspects of EF (set shifting, updating, and inhibition) as defined by Friedman and Miyake.^17^ Set shifting was measured with the number–letter task, where the subject is instructed to make a decision based on the number or the letter that appears on a screen by pressing one of two buttons.^59^ Updating was measured with an auditory N‐back, where the subject has to determine if the heard chord is the same or different compared with the previous (1‐back) or the chord before that (2‐back) by pressing one of two buttons.^60^ Inhibition was measured with the Simon task, for which the subject has to press the right button each time a red square appears, or the left button each time a blue square appears, irrespective of which side the square is presented on.^61^ As a measure of attention, we also used the Sustained Attention to Response Task,^62^ a computerized task with digits ranging from 1 to 9, where the subject has to respond to every other digit, except digit 3, by pressing a response button.

To determine behavioral recovery, the patients were assessed with the Behaviour Rating Inventory of Executive Function–Adult (BRIEF‐A) version.^63^ In the current study, we used three indices: behavioral regulation index (comprising the inhibit and self‐monitor scales), emotional regulation index (comprising the shift and emotional control scales), and metacognition index (comprising the remaining scales). These indices together formed the global executive composite index. In addition, we determined the effects of MBNR on everyday cognitive and emotional functioning as indicated by self‐report and caregiver‐report questionnaires on executive dysfunction, depression, and quality of life (QoL). Furthermore, we obtained subjective quantitative and qualitative feedback from the TBI patients and their caregivers regarding their experience of the intervention. To reduce potential bias introduced by the drop‐outs, an intention‐to‐treat (ITT) analysis with multiple imputation was used for the cognitive tests and questionnaire data. Per‐protocol analyses were also conducted as a way to assess the sensitivity of the ITT (for more details, see Ref. 53).

Our sample of moderate‐to‐severe TBI patients (*n* = 39; one patient was excluded due to intensive piano practice unrelated to the MBNR) had a mean age of 41.3 years (16 females and 23 males) and 8.9 months since injury on average, though the recruitment criteria included those sustaining an injury in the past 24 months (Table 1). The mean severity of the whole sample was moderate as indicated by the Glasgow Coma Scale (*μ* = 11.8) and the length of post‐traumatic amnesia (on average 1–7 days). The only clinical variable which differed between AB and BA groups concerned the etiology of the injury. However, this difference was not considered to be of clinical importance, particularly because the traffic‐related injuries involving higher energy and possibly a different recovery trajectory were evenly divided between the two groups. Regarding the musical background, the patients had on average between 4 and 6 years of experience either in singing, dancing, or instrument playing.

**TABLE 1:** Demographic, clinical, and musical background information

	All	AB	BA	Difference between groups, *p* value
**Demographic information**				
Age m (sd)	41.3 (13.3)	41.6 (14.7)	40.9 (12.0)	0.871 (*t*)
Gender (female/male)	16/23	10/10	6/13	0.242 (*χ* ^2^)
Handedness (right/left/both)	37/1/1	19/0/1	18/1/0	1.000 (F)
Education in years m (sd)	14.6 (3.2)	14.73 (2.8)	14.6 (3.6)	0.867 (*t*)
**Clinical information**				
GCS m (sd)	11.8 (4.2)	12.3 (3.6)	11.2 (4.7)	0.613 (*U*)
PTA classification^a^ m (sd)	2.1 (1.1)	1.9 (1.1)	2.3 (1.0)	0.280 (*t*)
Cause of injury (traffic related/fall/other)	16/15/8	8/11/1	8/4/7	0.022 (F)
Time since injury (months) m (sd)	8.9 (6.4)	8.6 (6.7)	9.2 (6.3)	0.772 (*t*)
Lesion laterality^b^ (left/right/both)	7/2/26	4/1/14	3/1/12	1.0 (F)
Contusion^c^ (yes/no)	23/15	13/6	10/9	0.508 (*χ* ^2^)
DAI^c^ (yes/no)	21/17	9/10	12/7	0.515 (*χ* ^2^)
Hemorrhages, bleeds, or ischemic injury^c^ (yes/no)	24/14	10/9	14/5	0.179 (*χ* ^2^)
GOSE^c^ m (sd)	5.2 (1.2)	5.0 (1.5)	5.5 (0.9)	0.192 (*t*)
NOS‐TBI^d^ m (sd)	2.0 (2.5)	2.2 (2.4)	1.8 (2.7)	0.385 (*U*)
BDI‐II m (sd)	14.2 (8.9)	15.8 (10.5)	12.3 (6.6)	0.229 (*t*)
**Musical background**				
Instrument playing (yes/no)	25/12	14/6	11/6	0.732 (*χ* ^2^)
Years of playing m (sd)	4.2 (8.4)	4.8 (10.3)	3.5 (5.4)	0.613 (*U*)
Singing (yes/no)	17/20	11/9	6/11	0.231 (*χ* ^2^)
Years of singing m (sd)	4.7 (9.9)	6.7 (12.7)	2.6 (5.3)	0.369 (*U*)
Dancing (yes/no)	20/17	12/8	7/10	0.254 (*χ* ^2^)
Years of dancing m (sd)	6.1 (10.8)	6.3 (10.6)	5.8 (11.4)	0.546 (*U*)

^a^ 1 = mild (< 24 h); 2 = moderate (1–7 days); 3 = severe (>7 days); 4 = very severe (>4 week).

^b^ Based on MRI findings.

^c^ Glasgow Outcome Scale Extended.

^d^ Neurological Outcome Scale for TBI.

The statistical analyses were done using a linear mixed model (LMM) for group AB and BA between TP1‐TP2, and a repeated measures ANOVA for group AB and LMM for group BA, with time (TP1, TP2, and TP3) as within‐subject factor.^53^ The significant group × time interactions were followed by pairwise comparisons. The results from the cognitive measures indicated that, compared to the BA group, general EF performance (FAB score) of the AB group improved over the intervention period, and the positive effect endured over the 6‐month follow‐up period (Figure 2A). In both groups, there was also improvement in set shifting (reduction of switching cost errors) during the intervention period compared to the control period (Figure 2B). No other cognitive measures showed a significant group × time interaction effect. Regarding the effects of MBNR on behavioral adjustment, we found a positive intervention effect on everyday EF functioning measured by the self‐reported BRIEF‐A behavioral regulation index in the AB group compared to the BA group, which was maintained in the 6‐month follow‐up (Figure 2C).^54^ Contrary to our expectations, we did not find any effect of the intervention on depression or QoL of the persons with TBI. However, the qualitative analysis revealed that elevated mood and importance of having meaningful activity were the central themes in the patients’ answers. Of note, TBI patients and their caregivers perceived the benefits in different areas in a similar fashion. Despite the lack of quantitative results, the qualitative feedback might be taken as evidence that MBNR contributed to the emotional well‐being on an individual and daily basis.

## BRAIN MORPHOMETRY CHANGES INDUCED BY MBNR

In addition to cognitive improvement in EF outcomes, in this RCT, we investigated whether MBNR could have a neuroprotective effect on the progressive brain atrophy that is commonly reported after TBI. Volumetric MRI measures, including voxel‐based^64^, ^65^, ^66^, ^67^ and deformed‐based morphometry,^67^, ^68^ have been used to identify this atrophy with sufficient statistical reliability and sensitivity to be applied in relatively small sample sizes.^69^ Crucially, this progressive loss of brain volume has been linked to cognitive impairments, including verbal reasoning and memory.^66^, ^67^ Hence, volumetric analyses provide surrogate measures of neurodegeneration that can potentially act as cost‐effective neuroimaging markers to evaluate the efficiency of clinical interventions. In fact, these measures are derived from T1‐weighted images, which are routinely and rapidly acquired in the clinical setting, and whose analysis can be automated with relative ease, especially for TBI patients without focal lesions. On this basis, previous studies with moderate‐to‐severe TBI patients in the chronic stage have found widespread gray matter volume reductions in frontal, temporal, occipital, and insula cortices bilaterally, as well as white matter volume loss in the corpus callosum, corona radiata, internal capsule, and brainstem.^67^ Importantly, gray matter volume changes have been reported after a music listening intervention with stroke patients,^70^, ^71^ pointing to the sensitivity of volumetric analysis to monitor structural plasticity changes induced by music‐based interventions in brain injury patients.

**FIGURE 4:**
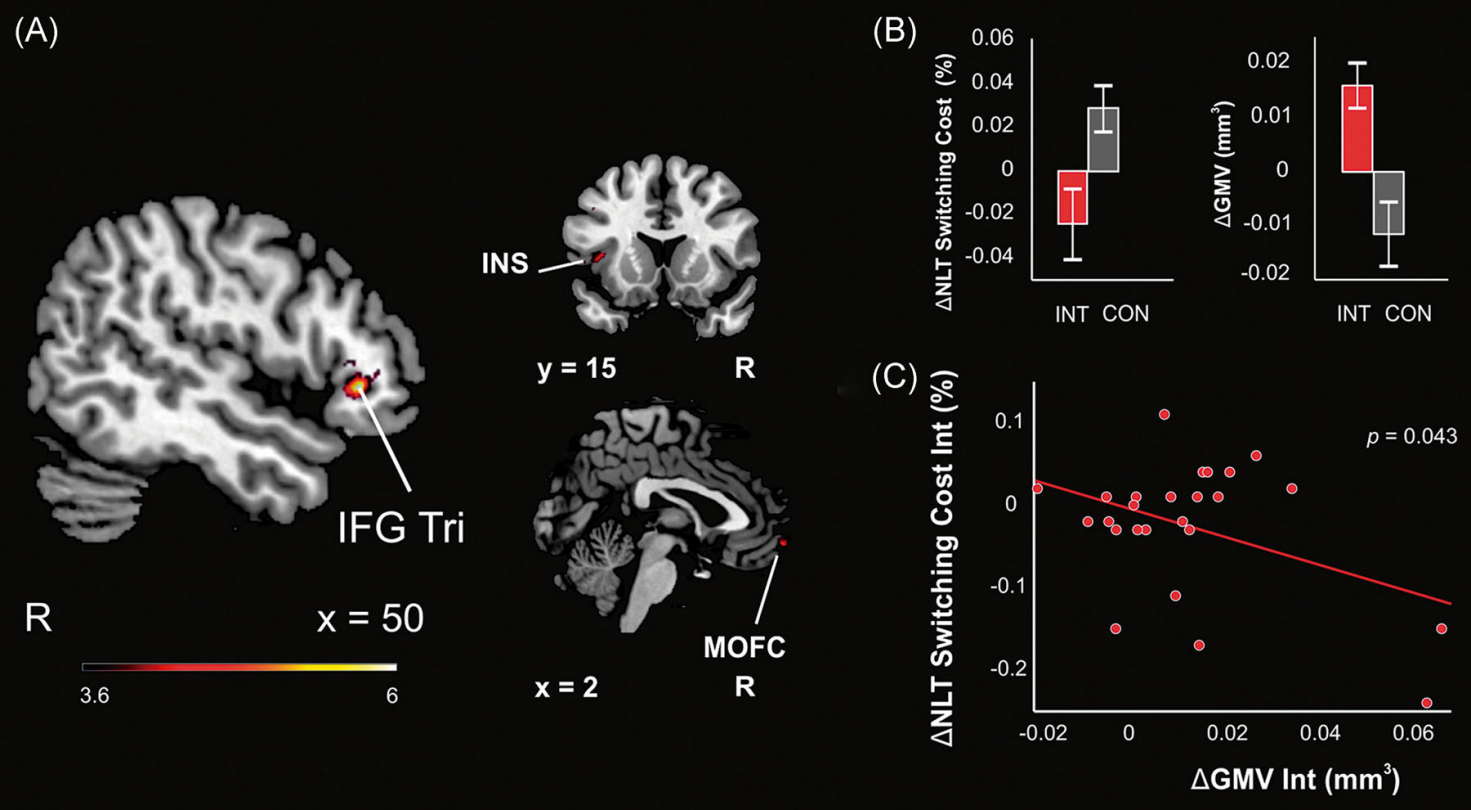
Changes during the intervention versus control period pooled across groups in 25 patients with TBI. (A) VBM results from the intervention versus control comparison (AB:TP2 > TP1 & BA:TP3 > TP2 vs. AB:TP3 > TP2 & BA:TP2 > TP1). The results are reported in MNI coordinates and at an uncorrected *p* < 0.001 threshold at the voxel level. The right IFG Tri cluster shown survived a *p* < 0.05 FWE‐corrected threshold at the cluster level. (B) Bar plots (mean SEM) for the Number–Letter Task switching cost score (errors, %) change (left) and the GMV change from the local maxima in the right IFG Tri cluster shown in A (right) during the intervention (INT) and control (CON) periods. (C) Pearson correlation between changes in the Number–Letter Task switching cost (errors, %) and in the GMV of the right IFG Tri cluster from A during the intervention period. Abbreviations: FWE, familywise error; GMV, gray matter volume; IFG Tri, inferior frontal gyrus triangular part; INS, insula; MNI, Montreal Neurological Institute; MOFC, medial orbitofrontal gyrus; R, right; SEM, standard error of the mean; TBI, traumatic brain injury; VBM, voxel‐based morphometry. Figure taken from Ref. 53

In our recent study,^53^ we found that gray matter volume in the right inferior frontal gyrus was significantly greater in both the AB and BA groups after the music intervention period, both when the groups were compared with each other across time (Figure 3) and when pooled together and compared with the control period (Figure 4A,B). Critically, this change was correlated with better set shifting abilities during the intervention period (Figure 4C). Other regions that most consistently showed an increase in gray matter volume after MBNR were the right middle frontal gyrus and the left superior frontal gyrus, cingulate cortex, insula, and cerebellum. One possible interpretation that fits well with the aforementioned spatial patterns of brain atrophy is that MBNR countered this neurodegeneration by providing environmental enrichment, which has been shown to protect against cognitive decline and atrophy after TBI in the chronic stage.^69^, ^72^

## RESTING‐STATE NETWORK PLASTICITY INDUCED BY MBNR

As alluded to above, TBI is often characterized by the presence of DAI and constitutes a pre‐eminent structural disconnection syndrome. As such, it allows to study the effects of long‐distance white matter tracts disruption on the functional connectivity (FC) of nodes in distributed brain networks and, crucially, their relationship with behavioral and clinical measures. A multimodal approach is important not only for assessing the potential value of structural and functional neuroimaging measures as sensitive markers for diagnosis but also to monitor the spontaneous recovery trajectory and the adaptive neural plasticity induced by the intervention in clinical trials. Further, compared to other brain injury disorders in which gray matter is also commonly affected (e.g., stroke), TBI can occur with no visible focal brain injury and be most likely restricted to the strained damage of axons caused by DAI, which makes it a paradigmatic case for analyzing the effects of damage to the structural connectome on brain FC. Since music production engages widespread brain regions, MBNR stands as a suitable rehabilitation strategy that may contribute to restore function by driving neural plasticity that balances the damaged system and helps it to regain its optimal functioning.

For this reason, we investigated the effects of MBNR on the resting‐state FC in large‐scale networks and their relationship with EF outcomes.^55^ More specifically, we analyzed FC patterns of four selected brain networks: the fronto‐parietal network (FPN), the dorsal attention network (DAN), the salience (SAL) network, and the default mode network (DMN), which were used as seeds to assess FC within them and between them and every other node in the resting‐state networks included in the CONN toolbox (for more details, see the Methods section in Ref. 55). The selection was based on brain networks whose constituent nodes have been previously found to play a functional role in the high‐level cognitive functions that are typically damaged after TBI, most frequently EF, attention, and working memory.^23^ This choice was further supported by the existing evidence showing that deficits in these cognitive domains are related to alterations in structural or FC after TBI. As mentioned above, it is possible to distill some commonalities in the abnormality patterns of large‐scale networks following TBI. For example, disruption of the structural connectivity in the SAL network has been associated with deficits in sustained attention and increased DMN activation, suggesting that SAL network integrity is needed to switch from an internally oriented focus of attention, as represented by the DMN, to a salient external stimulus, as supported by the SAL.^73^ The FC coupling between the SAL network and the DMN that allows for cognitive control has also been shown to be disrupted after TBI.^74^ Moreover, successful memory encoding has been associated with the DAN and ventral visual network activation in healthy participants, while active encoding was impaired in TBI patients, who showed abnormal activation within nodes of the DAN and regions regulating the DMN.^75^

**FIGURE 5:**
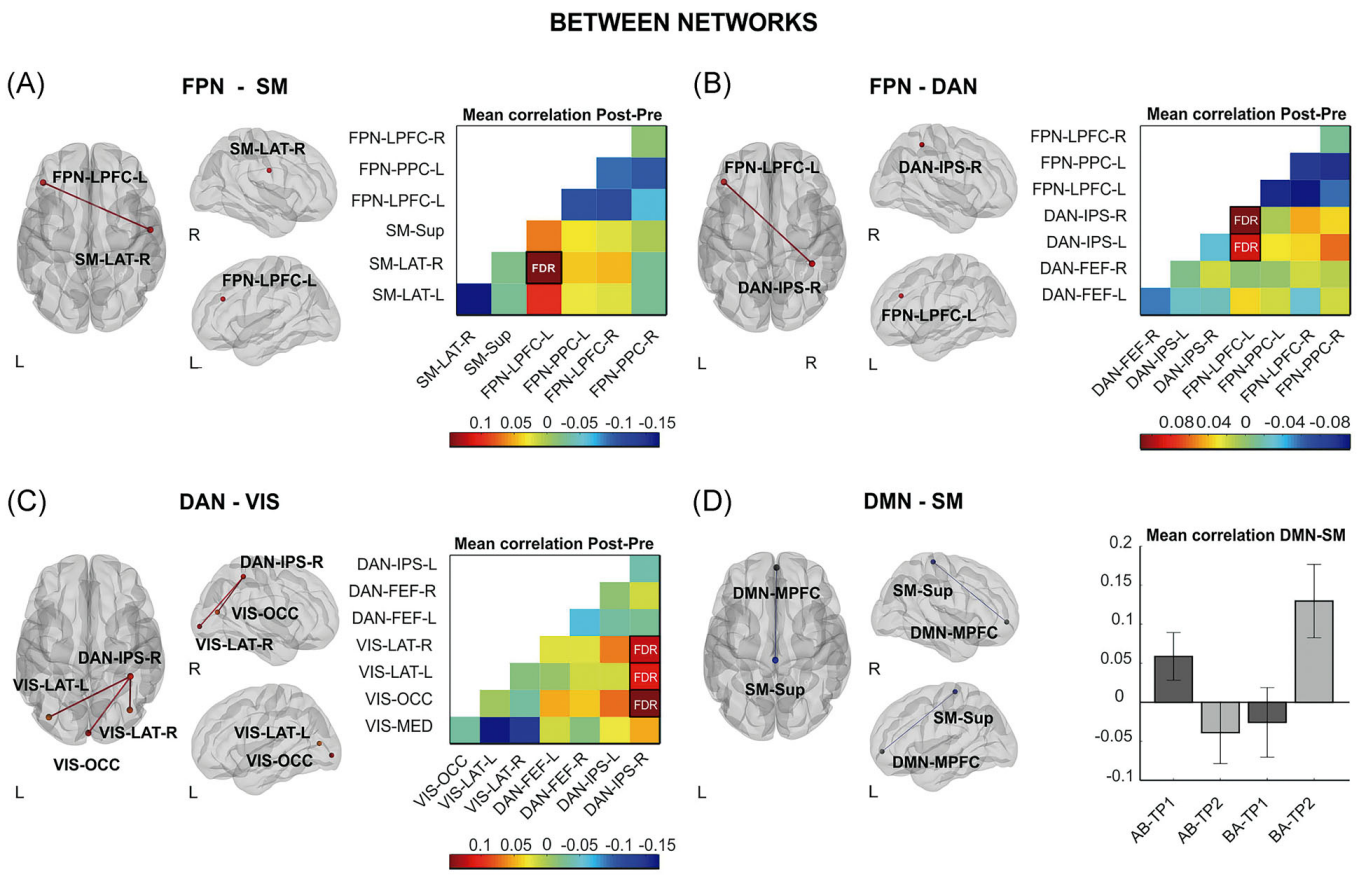
Changes in between‐network connectivity induced by the music‐based neurological rehabilitation. Nodes are overlaid on a rendered semitransparent brain generated using CONN. Connectivity matrices display the mean post‐ minus pre‐intervention (A–C) Fisher‐transformed Z‐score correlation values for each node. The bar plots (D) show the effect size of the AB>BA and TP2>TP1 interaction represented by the Fisher‐transformed Z‐score correlation values for each node. Abbreviations: DAN, dorsal attention; DMN, default mode network; FEF, frontal eye field; FPN, frontoparietal; IPS, intraparietal sulcus; L, left; LAT, lateral; LPFC, lateral prefrontal cortex; MED, medial; MPFC, medial prefrontal cortex; OCC, occipital; PCC, posterior cingulate cortex; PPC, posterior parietal cortex; R, right; SM, sensorimotor; Sup, superior; VIS, visual. Figure taken from Ref. 55

**FIGURE 6:**
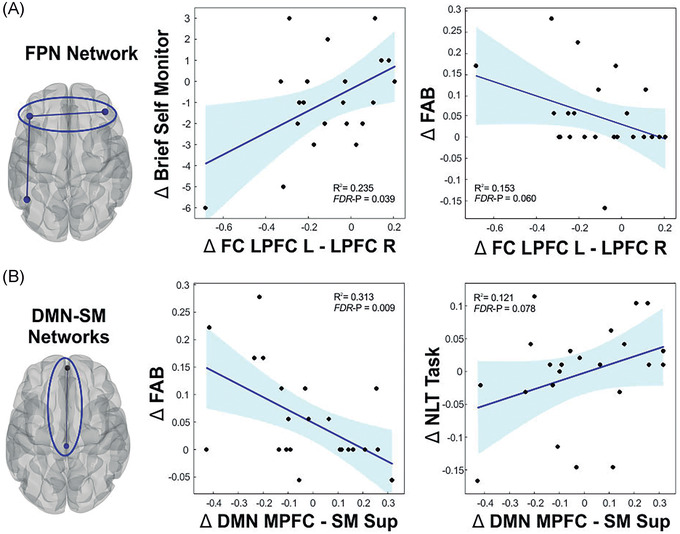
Within‐ and between‐network functional connectivity changes associated with cognitive recovery induced by the music‐based neurological rehabilitation. (A) The behavior and functional connectivity values are derived from the pre–post intervention comparison, which was significant for the within‐network connectivity changes in the FPN. (B) The behavior and functional connectivity values are derived from the AB > BA and TP2 > TP1 interaction, which was significant for the network connectivity changes between the DMN and the SM network. The scatter plots represent the bivariate Pearson correlation, and shaded areas represent the 95% CI prediction bounds. Abbreviations: DMN, default mode network; FPN, frontoparietal; L, left; LPFC, lateral prefrontal cortex; MPFC, medial prefrontal cortex; R, right; SM, sensorimotor; Sup, superior. Figure taken from Ref. 55

The results from our analysis revealed that MBNR induced an increase in the FC between the FPN and nodes of the sensorimotor (SM) network, as well as between the DAN and nodes of the visual network (Figure 5A–C). This effect could arise from the iterated interaction between the high‐level cognitive and perceptual systems recruited by music production, which are needed for self‐monitoring and motor adjustment based on SM and auditory feedback. The results of the increased interaction between the DAN and the visual (VIS) network are in keeping with the convergence of visual streams in the intraparietal sulcus within the DAN.^76^ We also found an increased coupling between the FPN and the DAN that might be related to improved regulation of perceptual attention, as suggested by recent work using meta‐analytic tools.^77^ On the other hand, the MBNR reduced the connectivity between the DMN and the SM network, which could reflect a reduction in the interference between the DMN and task‐driven activities mediated by the SM network (Figure 5D). The within‐network connectivity revealed specific nodes of the FPN and the SAL network, wherein coupling decreased in the pre‐ versus post‐intervention comparison. Importantly, the decrease in the FPN and DMN–SM connectivity was correlated with cognitive improvement in EF (Figure 6). In particular, those patients who showed a larger reduction in connectivity within the FPN network and between the DMN and the SM network after training exhibited greater improvement in general EF (higher FAB scores, both) and set shifting ability (i.e., less NLT errors; DMN‐SM FC decrease) along with greater reduction in executive deficits in self‐monitoring (i.e., smaller BRIEF‐A SM scores; FPN FC decrease). However, it is worth noting that the results from these correlations are based on a moderate sample of patients with some potential outlier values. Therefore, these results should be interpreted with caution and confirmed in future studies with larger sample sizes.

Considering what has been previously outlined so far in our review, it may seem counterintuitive that the MBNR induces a reduction in the FPN and that this is associated with improvement in EF. However, this finding is in alignment with the hyperconnectivity framework. This hyperconnectivity is defined as an increase in FC strength after TBI.^78^ According to the hyperconnectivity hypothesis, a major goal of the increase in FC following injury is to re‐establish network communication through network hubs in order to maximize information transfer and minimize cognitive impairments. However, as has been shown in a recent cost‐efficiency study,^79^ this is made at a higher metabolic cost for the network that can ultimately lead to the long‐term neurodegeneration patterns associated with TBI. The cost metric used by the authors includes the physical length as the Euclidean distance between nodes and, though it does not reflect a structural white matter connection directly, it is a good approximation for the cost that involves signaling through long distance connections in terms of metabolic energy and latency of neural transmission. This cost‐efficiency study demonstrated that there is a concomitant increase in FC and network cost at early stages of TBI, mostly due to increased medium‐range connections, whereas this cost is reduced at later stages during recovery. Although we did not compute measures of network cost, it is worthwhile noting the effects of reduced within‐network connectivity after the MBNR were located in frontal nodes, those showing the largest effect size for the increased network cost in Ref. 79.

In parallel to the aforementioned findings on the effects of MBNR on large‐scale resting‐state networks, our most recent work examining the structural connectome in the TBI patients from this RCT has revealed treatment‐induced changes in quantitative anisotropy in right frontal dorsal and projection pathways, as well as in the corpus callosum.^80^ Importantly, these changes were associated with better outcomes in the FAB. This suggests a wider picture where the changes in the white matter architecture could mediate to a certain extent the plasticity observed in the resting‐state FC and contribute to functional recovery.

## CONCLUSIONS AND FUTURE DIRECTIONS

In summary, in this RCT, we have demonstrated that MBNR is an efficient rehabilitative tool to ameliorate cognitive and behavioral symptoms after TBI, as well as to induce neural plasticity changes. The latter were identified structural and functionally via morphometric and resting‐state FC analyses. From our findings, it seems plausible that MBNR may have provided an enriched environment to slow down the brain volume loss reported in TBI, which is greater than that observed in healthy aging. Thus, we could speculate that MBNR acted as a neuroprotective element against brain atrophy and cognitive impairment. When examining the effects on resting‐state networks, we can draw three main conclusions: (1) it enhanced the coupling between high‐level cognitive (FPN, DAN) and perceptual (SM, VIS) networks, (2) reduced the connectivity within‐network in the FPN and between the DMN‐SM, and (3) the resting‐state FC changes were associated with improvement in EF. Taken together, these results suggest that resting‐state FC is a sensitive marker for the effects of MBNR on brain function.

However, there are still many questions that need to be addressed with regard to the mechanistic explanation for the efficacy of MBNR. For succinctness, we will focus on two of these questions in relation to some limitations in our resting‐state analysis. One of these limitations concerns the low number of nodes included in the network analysis, which was restricted to cortical regions. Therefore, our results are preliminary, and it would be relevant to replicate these findings with a fine‐grained brain parcellation scheme that includes subcortical regions. In particular, corticostriatal interactions could play a prominent role in the music‐induced benefits, as they are important for cognitive and motivational aspects that are both recruited during music making and targeted in TBI rehabilitation. In this sense, it is important to highlight that a disruption in the connectivity between the caudate and cortical regions in TBI has been linked to executive dysfunction together with increased levels of fatigue and apathy.^81^ Moreover, frontostriatal connections have been shown to be dependent on the levels of music reward sensitivity in the healthy population, suggesting that one mechanism for the rehabilitative effects of music might be experience‐dependent plasticity in these connections. The music‐induced dopamine release via the mesocortical and mesolimbic pathways could further contribute to the cognitive and emotional outcomes after TBI. From this perspective, music could provide a nonpharmacological dopaminergic stimulant that improves cognitive function as has been reported for methylphenidate in TBI patients.^82^

The second limitation is inherent to the static FC approach adopted for the resting‐state analysis, which overlooks the dynamic aspects of brain network function. Indeed, resting‐state brain networks are constantly reconfiguring over time to allow optimal information processing capabilities.^83^ In this vein, it is likely that the reduced executive functioning after TBI may be a consequence of reduced whole‐brain synchronization at global, network, and/or node level. Recently, a computational model framework has proposed that brain dynamics exhibit turbulent‐like behavior^84^ and that the optimal fitting of the brain model to the empirical data occurs when the turbulence amplitude levels as well as the integration/segregation capabilities are maximal. Therefore, future work should examine how turbulent‐like dynamics is disrupted after TBI, its relationship with behavioral measures, and its malleability with music‐based interventions. It is our hope that this computational modeling approach will provide important insights into the causal mechanisms underlying the neural plasticity induced by MBNR in individuals with TBI.

## COMPETING INTERESTS

The authors declare no competing interests.

## AUTHOR CONTRIBUTIONS

N.M.M. drafted the manuscript. All authors reviewed the manuscript.

### PEER REVIEW

The peer review history for this article is available at: https://publons.com/publon/10.1111/nyas.14800.
